# Correlation of gene polymorphisms of vascular endothelial growth factor with grade and prognosis of lung cancer

**DOI:** 10.1186/s12881-020-01030-0

**Published:** 2020-04-30

**Authors:** Changjiang Liu, Xuetao Zhou, Zefeng Zhang, Yang Guo

**Affiliations:** 1 Department of Thoracic Surgery, the Fourth Hospital of Hebei Medical University, 12 Jiankang Road, Shijiazhuang, 050000 China; 2 Department of Thoracic Surgery, the Third Hospital of Hebei Medical University, Shijiazhuang, China

**Keywords:** Vascular endothelial growth factor, Single nucleotide polymorphism, Tumor stage, Tumor prognosis

## Abstract

**Background:**

Vascular endothelial growth factor (VEGF) gene is highly polymorphic, and single nucleotide polymorphisms (SNP) of VEGF gene are associate with cancer prognosis. This study aimed to analyze the correlation of VEGF gene polymorphisms with grade and prognosis of lung cancer.

**Methods:**

A total of 458 Chinese patients with primary lung cancer were enrolled from September 2008 to October 2013. The genotypes of −2578C > A, −1154G > A, − 460 T > C, and + 405G > C were analyzed in white blood cells from patients using polymerase chain reaction based restriction fragment length polymorphism.

**Results:**

Our data showed that –1154G > A polymorphism was significantly associated with tumor stages, but all four tested VEGF gene polymorphisms had no significant effect on survival.

**Conclusions:**

VEGF polymorphisms may relate to stage of lung cancer in Chinese population.

## Background

Angiogenesis, the formation of new blood vessels from endothelial precursors, represents a crucial process in the growth and progression of numerous solid malignancies [1–3]. Therefore, the molecular mechanism of tumor-related angiogenesis has been of particular interest in the field of cancer research. The vascular endothelial growth factor A (VEGF-A) has been well identified as one of the key regulators of this process [4, 5]. Clinical studies have revealed that the VEGF-A pathway is associated with the angiogenesis grade and disease outcome for various solid tumors, including lung cancer [6, 7]. High VEGF-A in tumor tissue or in serum have proved to be related to advanced tumor stage and prognosis of non-small cell lung cancer [8–10]. In addition, VEGF-A expression has found to be markedly higher in adenocarcinomas than in squamous-cell carcinomas [8].

The VEGF-A gene is assigned to chromosome 6p21.1, and constitutes a highly polymorphic gene. Numerous single nucleotide polymorphisms (SNPs) have been recognized in the promoter, 5′-, and 3′-untranslated regions (UTR) of the VEGF-A gene [11, 12]. Importantly, clinical studies have revealed that VEGF SNPs are associated with the production and function of VEGF, and subsequently, have an impact on cancer risk and prognosis. For example, patients with lung cancer carrying − 2578 C/C, − 1154 A/A and G/A, and 450 G/G genotypes have low VEGF expression, whereas high VEGF expression was detected in samples from patients carrying the − 2578 C/A, − 1154 G/G, and 450 G/C genotypes [13]. In our previous study, we analyzed the relationship between four VEGF SNPs (including −2578C > A, −1154G > A, − 460 T > C, and + 405G > C) and risk of lung cancer, and found that C allele of + 405G > C was significantly associated with increased risk of lung cancer in males [14]. In this study, we studied the correlation of these SNPs with grade and prognosis of lung cancer.

## Methods

### Patients and samples

This study was approved by the Ethics Committee of the Fourth Affiliated Hospital of Hebei Medical University. Peripheral white blood cells were collected from 458 Chinese patients who had pathologically diagnosed as lung cancer at the Department of Thoracic Surgery, the Fourth Affiliated Hospital of Hebei Medical University from September 2008 to October 2013. The staging of lung cancer was assessed according to the WHO classifications. Information including age, sex, smoking history, site of disease, histopathological type, stage, and therapy was collected from all the patients. Written informed consent was obtained from all participants.

### Genotyping of VEGF gene polymorphisms

**Fig. 1:**
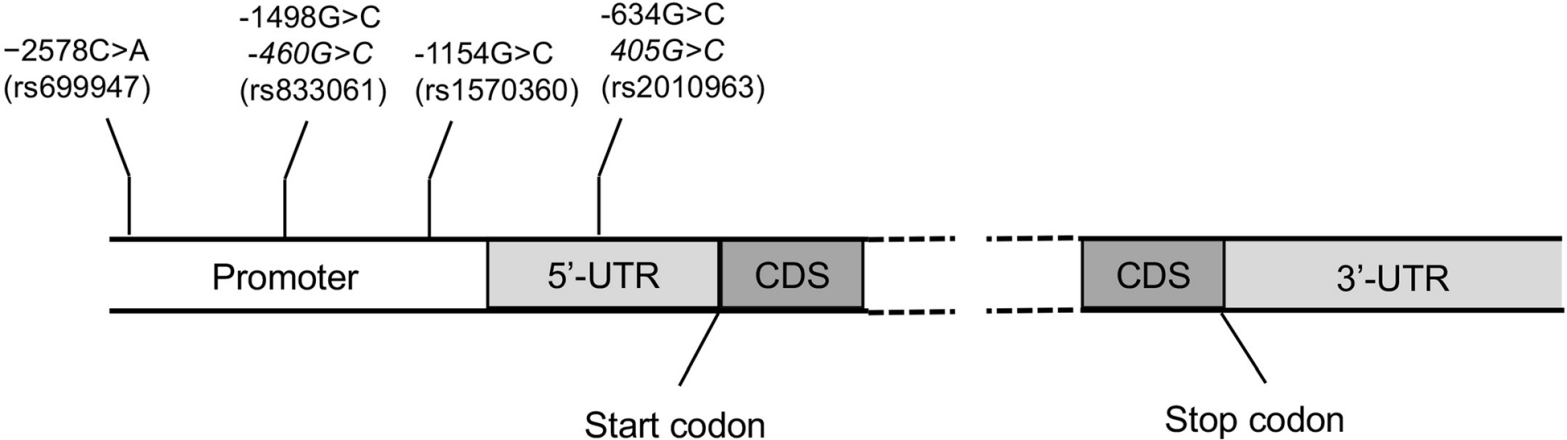
Position of VEGF SNPs relative to translation start site. Italic positions indicate alternative names

The genomic DNA was extracted from the peripheral white blood cells of patients using a genomic DNA purification kit (Promega). The genotypes of −2578C > A (rs699947), −1154G > A (rs1570360), − 460 T > C (rs833061), and + 405G > C (rs2010963) were then analyzed using polymerase chain reaction (PCR) based restriction fragment length polymorphism (RFLP) as described in our previous study. The position of 4 VEGF SNPs relative to translation start site was shown in Fig. 1. The primers used were: (1) −2578C > A F: 5′-CCTAGCACCTCCACCAAACCA-3′; −2578C > A R: 5′-CAGGGAACAAAGTTGGGGCTC-3′; 233 bp; (2) −1154G > A F: 5′-GGCGGATGGGTAATTTTCAGG-3′; −1154G > A R: 5′-TCCCCGCTACCAGCCGACTTT-3′; 236 bp; (3) − 460 T > C F: 5′-TGAATGG AGCGAGCAGCGTCT-3′; − 460 T > C R: 5′-CGTGCGGACAGGGCCTGAGA-3′; 236 bp; (4) +405G > C F: 5′-TGTGGATTTTGGAAACCAGCAGA-3′; +405G > C R:5′-CGGTGTCTGTCTGTCTGTCCG-3′; 234 bp. Restriction enzyme used were: (1) −2578C > A BglII; (2) −1154G > A BglII; (3) − 460 T > C Bs1236I; (4) +405G > C BglII. The expected result for each allele were: (1) −2578C > A: C/A genotype, 324 bp, 202 bp and 122 bp; C/C genotype, 324 bp; A/A genotype, 202 bp and 122 bp. (2) −1154G > A: G/A genotype, 164 bp and 150 bp; G/G genotype, 150 bp; A/A genotype, 164 bp. (3) − 460 T > C: T/C genotype, 175 bp and 155 bp; C/C genotype, 155 bp; T/T genotype, 175 bp. (4) +405G > C: G/C genotype, 304 bp, 193 bp and 111 bp; C/C genotype, 304 bp; G/G genotype, 193 bp and 111 bp.

### Statistical analysis

The genotypes for each SNP were analyzed as a three-group categorical variable. The differences in histopathological type, stage, or smoking year according to the four VEGF gene polymorphisms were compared using Chi square test or Fisher’s Exact Test, while the differences in overall survival (OS) were analyzed using log-rank tests. Cox proportional hazard regression model was used for the multivariate survival analyses, and the analyses were always adjusted for age, sex, smoking, histopathological type, stage, and therapy. Statistical analysis was done with SAS 9.3, and a cutoff *P* value of 0.05 was adopted for all analyses.

## Results

### Patient characteristics

A total of 458 patients with lung cancer were enrolled in this study. The age of the patients was 59.54 ± 9.30 years, and 320 (69.87%) patients were male, and 223 (48.69%) patients were smokers. The histopathological types of tumors included squamous cell carcinoma (*n* = 158, 34.50%), adenocarcinoma (*n* = 215, 46.94%), adenoacanthoma (*n* = 30, 6.55%), and others (*n* = 54, 12.01%). The tumor stages were as follows: stage I (*n* = 108, 23.58%), stage II (*n* = 163, 35.59%), stage III (*n* = 148, 32.31%), and stage IV (*n* = 49, 8.52%). During the 5 years of follow-up, 138 (30.13%) patients survived, and 300 (65.50%) patients died, and 20 (4.37%) patients were lost to follow-up.

### Genotype frequency

The four VEGF gene polymorphisms were successfully amplified in all cases. The frequencies of the genotypes were 5.24% (AA), 39.74% (AC), and 55.02% (CC) for VEGF -2578C > A; 2.18% (AA), 28.60% (AG), and 69.21% (GG) for VEGF -1154G > A; 5.46% (CC), 39.74% (CT), and 54.80% (TT) for VEGF –460 T > C; 19.00% (CC), 50.87% (CG), and 30.13% (GG) for VEGF +405G > C; all were consistent with Hardy-Weinberg equilibrium (*P* = 0.32, *P* = 0.50, *P* = 0.26, and *P* = 0.57 respectively).

### Genotype effects on tumor histopathological types and stages

Table 1 revealed that the proportions of all four VEGF gene polymorphisms were similar in different histopathological types of the tumor, which was consistent with our previous study. Table 2 showed that the –1154G > A polymorphism instead of other SNP genotypes was significantly associated with tumor stages (*P* = 0.042). Specifically, the proportion of A allele (AA and AG) was higher in the stage IV group (48.71%) compared to other stage groups (stage I, 34.26%; stage II, 26.38%; stage III, 28.38%). Smoking is the leading risk factor for lung cancer. Interestingly, Table 3 displayed that smoking status seemed to effect only on the distribution of –1154G > A polymorphism in patients (*P* = 0.060). The proportion of A allele (AA and AG) was higher in the patients with more than 25 years of smoking (38.60%) compared to non-smoking patients (26.39%) or patients with less than 25 years of smoking (25.00%).

**Table 1:** Frequency distribution of VEGF genotypes in different histopathological types of lung cancer

Variables	Squamous cell carcinoma	Adenocarcinoma	Adenoacanthoma	Others	***P*** value
	(*n* = 158)	(*n* = 215)	(*n* = 30)	(*n* = 55)	
−2578C > A (%)					0.964
AA	10 (6.33)	11 (5.12)	1 (3.33)	2 (3.64)	
AC	65 (41.14)	82 (38.14)	12 (40.00)	23 (41.82)	
CC	83 (52.53)	122 (56.74)	17 (56.67)	30 (54.55)	
−1154G > A (%)					0.814
AA	5 (3.16)	4 (1.86)	0 (0.00)	1 (1.82)	
AG	50 (31.65)	56 (26.05)	8 (26.67)	17 (30.91)	
GG	103 (65.19)	155 (72.09)	22 (73.33)	37 (67.27)	
−460 T > C (%)					0.969
CC	0 (6.33)	12 (5.58)	1 (3.33)	2 (3.64)	
CT	65 (41.14)	82 (38.14)	12 (40.00)	23 (41.82)	
TT	83 (52.53)	121 (56.28)	17 (56.67)	30 (54.55)	
+405G > C (%)					0.990
CC	29 (18.35)	42 (19.53)	7 (23.33)	9 (16.36)	
CG	81 (51.27)	108 (50.23)	14 (46.67)	30 (54.55)	
GG	48 (30.38)	65 (30.23)	9 (30.00)	16 (29.09)

**Table 2:** Frequency distribution of VEGF genotypes in different stages of lung cancer

Variables	I	II	III	IV	***P*** value
	(*n* = 108)	(*n* = 163)	(*n* = 148)	(*n* = 39)	
–2578C > A (%)					0.253
AA	6 (5.56)	6 (3.68)	11 (7.43)	1 (2.56)	
AC	47 (43.52)	59 (36.20)	55 (37.16)	21 (53.85)	
CC	55 (50.93)	98 (60.12)	82 (55.41)	17 (43.59)	
–1154G > A (%)					0.042
AA	1 (0.93)	6 (3.68)	2 (1.35)	1 (2.56)	
AG	36 (33.33)	37 (22.70)	40 (27.03)	18 (46.15)	
GG	71 (65.74)	120 (73.62)	106 (71.62)	20 (51.28)	
–460 T > C (%)					0.230
CC	7 (6.48)	6 (3.68)	11 (7.43)	1 (2.56)	
CT	47 (43.52)	59 (36.20)	55 (37.16)	21 (53.85)	
TT	54 (50.00)	98 (60.12)	82 (55.41)	17 (43.59)	
+405G > C (%)					0.846
CC	17 (15.74)	35 (21.47)	29 (19.59)	6 (15.38)	
CG	58 (53.70)	78 (47.85)	74 (50.00)	23 (58.97)	
GG	33 (30.56)	50 (30.67)	45 (30.41)	10 (25.64)

**Table 3:** Frequency distribution of VEGF genotypes in different smoking status of lung cancer

Variables	No	< 25	> = 25	***P*** value
	(*n* = 235)	(*n* = 52)	(*n* = 171)	
–2578C > A (%)				0.494
AA	11 (4.68)	2 (3.85)	11 (6.43)	
AC	88 (37.45)	19 (36.54)	75 (43.86)	
CC	136 (57.87)	31 (59.61)	85 (49.71)	
–1154G > A (%)				0.060
AA	3 (1.28)	1 (1.92)	6 (3.51)	
AG	59 (25.11)	12 (23.08)	60 (35.09)	
GG	173 (73.61)	39 (75.00)	105 (61.40)	
–460 T > C (%)				0.481
CC	11 (4.68)	2 (3.85)	12 (7.02)	
CT	89 (37.87)	19 (36.54)	74 (43.27)	
TT	135 (57.45)	31 (59.61)	85 (49.71)	
+405G > C (%)				0.493
CC	46 (19.57)	8 (15.38)	33 (19.30)	
CG	125 (53.19)	29 (55.77)	79 (46.20)	
GG	64 (27.24)	15 (28.85)	59 (34.50)	

### Genotype effects on survival

All four tested VEGF gene polymorphisms had no significant effect on survival in the univariate or multivariate analysis (Table 4). As the four VEGF gene polymorphisms are in linkage disequilibrium, haplotype analyses were conducted to assess the combined effect of the four SNPs on lung cancer survival (Table 5). Six common haplotypes (CGTG, 26.55%; AACG, 5.65%; AGCG, 4.37%; AGTG, 4.83%; CGCG, 4.99%; CGTC, 35.36%) were inferred. Unfortunately, no significant independent association was found for haplotypes and survival.

**Table 4:** Survival analysis according to four VEGF gene polymorphisms

Variables	OR (95%CI)	***P*** value
–2578 C > A (%)		0.997
AA vs CC	0.525 (0.217–1.270)	0.156
AC vs CC	0.965 (0.633–1.471)	0.304
–1154 G > A (%)		0.938
AA vs GG	0.679 (0.187–2.460)	0.568
AG vs GG	0.974 (0.621–1.529)	0.66
–460 T > C (%)		0.998
CC vs TT	0.471 (0.199–1.117)	0.091
CT vs TT	0.952 (0.624–1.453)	0.234
+405 G > C (%)		0.918
CC vs GG	1.402 (0.765–2.567)	0.267
CG vs GG	1.068 (0.677–1.686)	0.626

**Table 5:** Survival analysis according to common haplotypes of four VEGF gene polymorphisms

Haplotypes (−2578C > A/−1154G > A/−460 T > C/+ 405G > C)	Haplotype frequency (%)	OR (95%CI)	P value
CGTG	26.55	1	
AACG	5.65	0.870 (0.753–1.004)	0.058
AGCG	4.37	0.916 (0.776–1.081)	0.300
AGTG	4.83	0.922 (0.797–1.067)	0.276
CGCG	4.99	0.905 (0.782–1.047)	0.179
CGTC	35.36	1.021 (0.946–1.101)	0.590

All those frequency < 0.03 were ignored in the analysis

## Discussion

VEGF is a highly polymorphic gene, and numerous evidence has revealed that VEGF SNPs are associated with cancer risk and prognosis. In our previous study, we found that C allele of VEGF +405G > C genotype was significantly associated with increased risk of lung cancer [14]. In this study, we found that VEGF -1154G > A polymorphism was significantly associated with tumor stages, but all four tested VEGF gene polymorphisms had no significant effect on survival.

It is reported that the -1154A/A and G/A genotype were linked with low VEGF expression, and the -1154A/A polymorphism also related with poor vascularization in patients with lung cancer [13]. In this study, our data showed that -1154G > A polymorphism was significantly associated with tumor stages. Specifically, the proportion of A allele (AA and AG) was higher in the stage IV group compared to other stage groups. This result seemed to contradict with the established conclusion, because A allele in -1154G > A is believed to associate with low VEGF expression and poor vascularization. However, it is also possible that A allele might slow down cancer progression and thus patients become aware of the disease later, when their cancer progresses further. In addition, we found that there was a strong trend between presence of A allele for -1154G > A and smoking for > 25 years (*p* = 0.06). It could be that cancer progresses more slowly in these patients and they develop disease after longer period of smoking. Heist et al reported that patients with lung cancer carrying the variant C allele of the VEGF +405G > C polymorphism had significantly improved survival [15]. Our previous study showed that C allele of +405G > C was significantly associated with increased risk of lung cancer in males [14]. However, no association between the +405G > C polymorphism and survival was found in this study. In addition, VEGF −2578C > A and − 1154G > A were found to have a marked impact on the survival of breast cancer [16, 17], but our study failed to find any association between these two SNPs and the survival of lung cancer. It is generally accepted that the ethnicity of study subjects is a crucial factor for interpreting genetic polymorphism studies [18]. The population of our study is Chinese, which is different from the cited studies. Therefore, the inconsistent findings between our study and other studies might attribute to the ethnic differences. Finally, it is worthy to point out that -460C/C polymorphism seemed to relate with survival (OR, 0.471; 95% CI, 0.199–1.117) but the difference did not reach significance (*p* = 0.091). This OR could have been more significant in a larger cohort of patients. Importantly, there is also a trend for association between AACG haplotype and survival (*p* = 0.058; OR, 0.870; 95% CI, 0.753–1.004). This is interesting because these patients have A allele for -1554G > A and G allele for +405G > C that are associated with lower vascularization and lower risk of developing lung cancer, respectively.

## Conclusions

Our data revealed that VEGF polymorphisms may relate to stage but not survival of lung cancer in Chinese population. Our findings should spur the interest of additional investigation of gene polymorphisms in VEGF as well as the other angiogenesis pathway associated with lung cancer risk and outcomes.

## Data Availability

All data generated or analyzed during this study are included in this published article.

## Abbreviations

VEGF-A: Vascular endothelial growth factor A
SNP: Single nucleotide polymorphism
UTR: Untranslated regions
PCR: Polymerase chain reaction
RFLP: Restriction fragment length polymorphism
OS: Overall survival
